# Supplementary material for: Stable excitatory-inhibitory synapse balance despite dynamic turnover
Source: eLife. 2026 Jul 27;14:RP107635. doi: 10.7554/eLife.107635 (PMC13405638; doi:10.7554/eLife.107635)
Supplement: Peer review history

# Editor report: eLife Assessment

**DOI:** 10.7554/eLife.107635.3.sa0

**Authors:** Hui-Li Wang

**Affiliations:** Hefei University of Technology China

**Keywords:** Convincing, Important

This **important** study establishes a robust live-imaging toolkit to characterize excitatory and inhibitory synaptic dynamics during neuronal development, advancing our mechanistic understanding of synaptic homeostasis and neural circuit maturation. The core findings clarify how stable E/I balance is maintained despite persistent synaptic turnover, with broad implications for developmental neurobiology and neurodevelopmental disorders. The methodology and quantitative data are **convincing** and well validated, and this work represents a significant advance that will be of significant interest to researchers in synaptic biology, cell imaging and neuroscience.

# Referee report: Reviewer #1 (Public review)

**DOI:** 10.7554/eLife.107635.3.sa1

[Editors' note: all three reviewers confirm that all initial concerns have been fully resolved through comprehensive revisions and supplementary analyses.]

Summary:

By imaging the dynamics of synaptic proteins in cultured neurons, this study presents significant findings regarding the dynamics of excitatory and inhibitory synaptic proteins during development. The evidence shows that the ratios of excitatory and inhibitory synaptic proteins are stable during synapse development. This discovery advances our understanding of the complex mechanisms governing synapse formation. The strength of the evidence is robust, as it is supported by a combination of biological assays and endogenous labeling.

Strengths:

This research sheds light on the dynamics of the excitatory and inhibitory synapses during development. It is crucial to understand that while excitatory synapses and inhibitory synapses are developed independently, the ratio of their number is relatively stable during development, maintaining a stable excitatory/inhibitory ratio.

Important findings and implications in the research include:

(1) Persistent Synapse Dynamics: Excitatory and inhibitory synapses remain highly dynamic even in mature neurons (DIV12-14), challenging the dogma that synaptic structures are stable after the synaptogenesis stage.

(2) Maintained E/I Balance: Despite ongoing synapse turnover (formation/elimination) and presynaptic terminal reduction, the overall density and ratio of excitatory-to-inhibitory synapses remain relatively stable during circuit maturation (Figure 7).

(3) Developmental Shifts: While presynaptic compartments decrease over time, postsynaptic sites increase, suggesting independent regulation of pre- and postsynaptic elements within a stable E/I framework.

Weaknesses:

This study focuses on specific synaptic proteins within synapses, which may not fully represent the dynamics of other synaptic machinery; also, whether similar observations exist in vivo is still unknown. Further research is needed to explore the implications of these findings in more complex neuronal environments.

Comments on revised version:

The authors have addressed all my questions/comments. No further questions for this manuscript.

# Referee report: Reviewer #2 (Public review)

**DOI:** 10.7554/eLife.107635.3.sa2

Summary:

The Garbett et al. identified a critical need to begin to understand the interplay between the assembly, maturation, and elimination of excitatory and inhibitory synapses. They also detail the lack of reliable tools to address this gap in knowledge. Here, the authors developed synaptic reporters expressed by lentiviruses (mClover3-Homer1c, HaloTag-Syb2, and tdTomato-Gephyrin). They combined these reporters with resonance scanning confocal imaging to measure synapses over a 15-hour period during neuron development and in mature neurons in primary hippocampal cultures. Using these reporters in the same neuron, the authors compared the ratios of postsynaptic excitatory and inhibitory specializations that co-localize with presynaptic terminals during development and in mature neurons and found that they are stable across time points. Finally, the authors developed CRISPR/Cas9 tools (TKIT) to knock-in endogenous fluorescent tags (GFP/tdTomato-Gephyrin) or epitope tags (HA-Bassoon and HA-Homer1) to begin to study synapse dynamics using endogenous proteins. I believe this paper highlights an important gap in knowledge and begins to offer methodologies to determine the dynamic coordination between excitatory and inhibitory synapses.

Strengths:

(1) The experiments are well-designed and carefully controlled.

(2) The authors carefully validated the reporter and TKIT constructs.

(3) The authors provide strong proof-of-principle for the use of the reporter constructs to track synapse formation, maintenance, and elimination over a 15-hour period.

(4) Ingenious use of technologies (reporters, TKIT, and resonance scanning confocal microscopy) to develop a platform for future studies of synapse dynamics.

(5) Strong evidence supporting that the ratio of excitatory and inhibitory synapses (those that oppose syb2) stays constant through development.

Overall, this is a well-executed study that develops tools to simultaneously image excitatory and inhibitory synapse dynamics and represents an important first step to address the fundamental question regarding the coordination between these two types of synapses.

Comments on revised version:

The authors addressed all my questions and comments. Their edits have made this paper significantly stronger. I believe that this is an important paper for the field.

# Referee report: Reviewer #3 (Public review)

**DOI:** 10.7554/eLife.107635.3.sa3

In the present study, the authors describe the development of new tools and imaging strategies to assess the concomitant development of excitatory and inhibitory synapses in dissociated neuron cultures. To this end, they generate fluorescently tagged constructs of excitatory and inhibitory synapse marker proteins using either conventional overexpression or CRISPR-based strategies. They then image these marker proteins over a timespan of 15 hours to assess synaptic dynamics at different developmental timepoints. Based on their data, they conclude that excitatory and inhibitory synapse development occur in concert to maintain a functional balance despite individual synapse turnover.

Overall, this study addresses an interesting question, i.e., the interplay between the development of excitatory and inhibitory synapses, which has important implications, particularly for neurodevelopmental disorders in which the balance of excitation and inhibition is disrupted. The experiments are technically solid and well-executed, and the individual images are highly compelling.

Comments on revised version:

The authors have fully addressed my concerns, and this is now a strong manuscript for the synaptic field.

# Author comment: Author response

**DOI:** 10.7554/eLife.107635.3.sa4

**Authors:** Krassimira A Garbett, James P Allen, Jaybree M Lopez, Cassandra M Smith, Richard C Sando

**Affiliations:** Vanderbilt University Nashville United States; Vanderbilt University Nashville United States; Vanderbilt University Nashville United States; Vanderbilt University Nashville United States; Vanderbilt University Nashville United States

The following is the authors’ response to the original reviews.

**eLife Assessment**

In this valuable study, the authors developed long-term imaging tools to simultaneously monitor the temporal and spatial dynamics of excitatory and inhibitory synapses and reported that excitatory and inhibitory synapses need to develop synergistically during synaptogenesis to maintain balance. While the analysis and quantification of the imaging data are incomplete, there is convincing evidence that the developed tools are feasible. If these tools can function stably in vivo, their applications will be much broader.

We have completely overhauled our analysis and quantification methods and generated custom-made drift correction and tracking pipelines. Also, we have tested these tools ex vivo.

**Public Reviews:**

**Reviewer #1 (Public review):**

Summary:

By imaging the dynamics of synaptic proteins in cultured neurons, this study presents significant findings regarding the dynamics of excitatory and inhibitory synaptic proteins during development. The evidence shows that the ratios of excitatory and inhibitory synaptic proteins are stable during synapse development. This discovery advances our understanding of the complex mechanisms governing synapse formation. The strength of the evidence is robust, as it is supported by a combination of biological assays and endogenous labeling.

Strengths:

This research sheds light on the dynamics of the excitatory and inhibitory synapses during development. It is crucial to understand that while excitatory synapses and inhibitory synapses are developed independently, the ratio of their number is relatively stable during development, maintaining a stable excitatory/inhibitory ratio.

Important findings and implications in the research include:

(1) Persistent Synapse Dynamics: Excitatory and inhibitory synapses remain highly dynamic even in mature neurons (DIV12-14), challenging the dogma that synaptic structures are stable after the synaptogenesis stage.

(2) Maintained E/I Balance: Despite ongoing synapse turnover (formation/elimination) and presynaptic terminal reduction, the overall density and ratio of excitatory-to-inhibitory synapses remain relatively stable during circuit maturation (Figure 7).

(3) Developmental Shifts: While presynaptic compartments decrease over time, postsynaptic sites increase, suggesting independent regulation of pre- and postsynaptic elements within a stable E/I framework.

We thank the Reviewer for their positive feedback and careful review of our study.

Weaknesses:

This study focuses on specific synaptic proteins within synapses, which may not fully represent the dynamics of other synaptic machinery; also, whether similar observations exist in vivo is still unknown. Further research is needed to explore the implications of these findings in more complex neuronal environments.

We also thank the Reviewer for their insights and suggestions. We have added discussion of this important point to the Discussion section. Furthermore, we have tested the applicability of our tools ex vivo (new Figures 1, 4, and 6). While using these tools in vivo for live imaging is the eventual goal, we started in a reduced culture system given the relative simplicity. Our current study now provides a framework for future experiments applying these approaches in more complex in vivo systems.

**Reviewer #2 (Public review):**

Summary:

The Garbett et al. identified a critical need to begin to understand the interplay between the assembly, maturation, and elimination of excitatory and inhibitory synapses. They also detail the lack of reliable tools to address this gap in knowledge. Here, the authors developed synaptic reporters expressed by lentiviruses (mClover3-Homer1c, HaloTag-Syb2, and tdTomatoGephyrin). They combined these reporters with resonance scanning confocal imaging to measure synapses over a 15-hour period during neuron development and in mature neurons in primary hippocampal cultures. Using these reporters in the same neuron, the authors compared the ratios of postsynaptic excitatory and inhibitory specializations that co-localize with presynaptic terminals during development and in mature neurons and found that they are stable across time points. Finally, the authors developed CRISPR/Cas9 tools (TKIT) to knock-in endogenous fluorescent tags (GFP/tdTomato-Gephyrin) or epitope tags (HA-Bassoon and HAHomer1) to begin to study synapse dynamics using endogenous proteins. I believe this paper highlights an important gap in knowledge and begins to offer methodologies to determine the dynamic coordination between excitatory and inhibitory synapses.

Strengths:

(1) The experiments are well-designed and carefully controlled.

(2) The authors carefully validated the reporter and TKIT constructs.

(3) The authors provide strong proof-of-principle for the use of the reporter constructs to track synapse formation, maintenance, and elimination over a 15-hour period.

(4) Ingenious use of technologies (reporters, TKIT, and resonance scanning confocal microscopy) to develop a platform for future studies of synapse dynamics.

(5) Strong evidence supporting that the ratio of excitatory and inhibitory synapses (those that oppose syb2) stays constant through development.

We thank the Reviewer for their positive assessment of our study.

Weaknesses:

Overall, this is a well-executed study that develops tools to simultaneously image excitatory and inhibitory synapse dynamics and represents an important first step to address the fundamental question regarding the coordination between these two types of synapses.

Minor weaknesses of the manuscript include:

(1) The lack of a characterization of endogenous Homer1-positive excitatory synapses using TKIT.

We attempted to perform live imaging of endogenous Homer1-positive synapses using the TKIT approach by tagging endogenous Homer1 with mClover3 but encountered low signal/noise while live imaging. This prompted us to focus our current study on live imaging endogenous Gephyrin. Future studies using more robust tags (e.g. StayGold, HaloTag) for TKIT tagging of endogenous Homer1 will likely help circumvent this issue.

(2) Discussion about other approaches to study excitatory and inhibitory synapses using endogenous proteins (e.g., intrabodies - FingR or nanobodies) should be included.

This important point was also raised by other Reviewers. We have now significantly expanded the Discussion section, including discussion of this point.

(3) The activity state of a neuron and/or a synapse might alter the dynamic properties (formation, maintenance, and/or elimination). A discussion on whether the overexpression of Homer1 and/or gephyrin might alter synapse/neuron activity would provide greater interpretability of the results. A discussion of the potential limitations and benefits of the reporter and TKIT approaches would be beneficial.

We agree and have added discussion of these points to the Discussion section.

(4) A description and interpretation of the computational approach to calculate particle tracking would be helpful. I found that particle tracking figures, while elegant, are difficult to interpret.

As discussed in more detail below, we have generated drift correction and particle tracking approaches for the revised manuscript. We now elaborate on these new approaches in the paper.

We thank the Reviewer again for their very helpful input and suggestions.

**Reviewer #3 (Public review):**

In the present study, the authors describe the development of new tools and imaging strategies to assess the concomitant development of excitatory and inhibitory synapses in dissociated neuron cultures. To this end, they generate fluorescently tagged constructs of excitatory and inhibitory synapse marker proteins using either conventional overexpression or CRISPR-based strategies. They then image these marker proteins over a timespan of 15 hours to assess synaptic dynamics at different developmental timepoints. Based on their data, they conclude that excitatory and inhibitory synapse development occur in concert to maintain a functional balance despite individual synapse turnover.

Overall, this study addresses an interesting question, i.e., the interplay between the development of excitatory and inhibitory synapses, which has important implications, particularly for neurodevelopmental disorders in which the balance of excitation and inhibition is disrupted. The experiments are technically solid and well-executed, and the individual images are highly compelling.

We thank the Reviewer for their positive assessment of our study.

However, a number of aspects remain to be addressed in order for the study to support the claims made by the authors. First, the novelty aspect of the development of the fluorescently tagged synaptic proteins is unclear, since reporters of this nature are in routine use in many labs. Second, the analysis of the acquired images often seems incomplete, with only example images but no quantification shown, or the distinction between spatial and temporal dynamics appearing unclear. Third, given this incomplete analysis, the interpretations of the authors are not always convincingly supported by the data presented. In conclusion, substantial improvements are required to render the main messages of the study clear and compelling.

We agree and have incorporated all of the Reviewer’s suggestions in the revised manuscript (please see below).

**Recommendations for the authors:**

**Reviewer #1 (Recommendations for the authors):**

This is an interesting study. This reviewer has the following questions/comments for the authors:

(1) Please provide evidence that the gRNAs targeting each gene of synaptic protein have no offtarget effects.

We now include analysis of off-target effects for the TKIT tools (new Figure S6).

(2) While structural E/I balance is shown, functional electrophysiological validation (e.g., mEPSC/mIPSC ratios) is absent. It is interesting to know whether the balanced functional structural changes translate to functional?

We thank the Reviewer for this insightful suggestion and now include these recordings in the revised paper (new Figure 8).

(3) In lines 217-218, please define thresholds for "stable" vs. "dynamic" puncta (e.g., temporal and spatial criteria).

We more clearly define our categorization parameters (e.g. new Figure 2).

(4) In Figure 5B: The low co-localization between endogenously tagged Bassoon and antibodystained Bassoon is likely due to the low TKIT efficiency. Quite a few HA-tagged Basson signals are insensitive to Basson-antibody. The authors are suggested to explain those.

We thank the Reviewer for identifying this and add discussion to the Results section.

(5) For the data analysis. If each n represents an independent neuronal culture, should the authors are suggested to provide the number of neurons/dendrites analyzed for each independent culture?

We have added these important details to the manuscript.

(6) Regarding the title, the author used the term "coordinated dynamics". This reviewer finds it is a bit over-claim because the stable ratios of the number of excitatory synapses and inhibitory synapses are likely an association, not actively "coordinated". I suggest that the authors rephrase this.

We agree that we cannot argue that excitatory and inhibitory synapses are causally coordinated in our current study. Their levels are likely associated by either association or direct coupling, which we now discuss further in the first paragraph of the Discussion. We have rephrased the title accordingly.

**Reviewer #2 (Recommendations for the authors):**

I have only minor suggestions that I think will improve the manuscript:

(1) Please define Syn1/2 on line 129.

We have defined this in the revised paper.

(2) For Figures 2B, C, and 4B, C: are the puncta in panel C from the dendrites in panels B? If so, it would be helpful to identify the ROIs selected in panels C.

We now include this in new Figure 2.

(3) For the particle tracking figures, while the ability to track all synaptic puncta is very impressive, it is sometimes difficult to clearly track the lifespan of a synaptic puncta from the current figures. I believe that it would be helpful if the authors selected specific examples of synapses formed, maintained, and eliminated.

We agree and now include more examples.

(4) I believe that more detail about the computational approach and analysis for the particle tracking (Figs 2E and 4E) would help the interpretability of the figure.

This important point was also raised by the other Reviewers. We generated custom tools during the revision that significantly expand the capabilities of our tracking approaches and more clearly describe them in the revised manuscript.

(5) Similar to the rigorous gephyrin TKIT analysis (Fig. 6), did the authors perform a similar analysis for Homer1c TKIT? This might be valuable to confirm that overexpression of the Homer1 reporter does not indirectly alter synapse dynamics.

We attempted to perform live imaging of mClover3 TKIT-tagged endogenous Homer1 but encountered low signal/noise with live imaging. We now add discussion that optimization of more robust tags (e.g. StayGold, HaloTag) will likely be necessary for live imaging of different target proteins.

(6) The tools developed by Garbett et al. have the potential to be broadly utilized in the field to provide new insight into the coordination of excitatory and inhibitory synapses. It would thus be helpful for the authors to include a discussion about the strengths and limitations of the reporter and TKIT methods relative to other approaches used to live image synapses (e.g., intrabodies (FingR and nanobodies)).

We have now significantly expanded the Discussion to include these important points.

(7) In the discussion, can the authors elaborate on whether it is experimentally feasible to apply their TKIT labeling of gephyrin and Homer1c in the same neuron to assess the endogenous excitatory and inhibitory synapse dynamics from the same neuron?

We have added discussion of this point and also proof-of-concept data supporting tagging of two postsynaptic targets within the same neuron (new Figure S5D).

**Reviewer #3 (Recommendations for the authors):**

(1) While the new tools described in the current manuscript can undoubtedly be used for the described purposes, the novelty of these tools is unclear to me. Viral vectors expressing fluorescently tagged versions of Homer1, synaptobrevin, and gephyrin are commercially available, e.g., via Addgene, and they are in routine use in many labs. CRISPR-mediated strategies for this purpose have also been previously reported (e.g., Willems et al. 2020, PLOS Biology; Fang et al. 2021, eLife). It is not clear to me how the tools reported here present a significant improvement over existing resources, other than that they use different fluorescent tags. If this aspect is a central part of the current manuscript, it should be expanded on in the discussion, including a direct comparison with available tools to highlight the novel aspects.

We agree and have significantly expanded the Discussion to include these important points. Also, rather than argue that our tools are superior to pre-existing approaches, we adjust the text to argue that our tools and analytical approaches have been designed and optimized for the purposes we apply them to.

(2) In addition to generating new tagged constructs, the authors also state that they have developed new imaging and analysis strategies to facilitate long-term assessment of synaptic dynamics. However, in many figures, they present only sample images, with little quantification to allow assessment of the wider relevance of the imaged synapses. For example, in Figures 2C and 4C, they present one example each of, e.g., a stable, nascent, transient, or eliminated synapse. However, they do not provide any quantification on how frequently any of these events occur, or whether they can be reliably quantified at all. These quantifications (i.e., percentage of each event type across a large population of synapses) would be necessary and should be added to demonstrate that this tool can be used for more than single example images.

We have generated custom-made drift correction and particle tracking approaches for the revised manuscript. Based on the reviewer’s suggestion, we have quantified the relative frequencies of stable, nascent, transient, and eliminated synapses (Fig 2B-G, Fig3A-F, Fig 5A-F, Fig 7B-C). These metrics greatly enhance the biological interpretation of our results. We have also added a supplemental movie with an example image with corresponding categorized tracks for each puncta type (Movie S3)

(3) The authors do present an automated visual representation of spatial track length across the neuron, e.g., in Figure 2E and 4E, although this is also not quantified. Moreover, the track lengths appear surprisingly short, despite the authors' claims that their analyses 'highlight the dynamic nature of excitatory synapses over these timescales'. It is not clear to me whether these short tracks are more than just jitter, either in the synapses themselves or in the images due to technical limitations. E.g., in panel 2E, I see very few examples in which the track is not simply centered around one point, but actually expands over a distance. Quantification of the distance between start and end points of the tracks would be important to support the claim that these synapses are dynamic in terms of spatial translocation (if that is what the authors meant). Or if the 'dynamic nature' of the synapses referred to temporal dynamics, it is unclear to me how this information can be gained from the represented tracks.

We thank the reviewer for these excellent points. To accurately access spatial motion, we drift-corrected our images with a custom correction algorithm to eliminate stage or microscope drift as a source of contaminating motion (See Methods, Movie S2), in addition to collecting time-lapse imaging with Nikon perfect focus. We noticed heterogeneity in our cultures such that some areas contained very mobile neurites, while other remained stationary (Fig. S1). We binned movies into either moving or still neurites and assessed spatial metrics as suggested (Fig. S1A). Consistent with our binning, puncta on moving neurites showed larger net displacement (distance between start and end points), but puncta on still neurites also showed ~1 µm net displacement (Fig. S1D). We also quantified puncta speed and found that puncta on moving neurites generally moved faster (Fig. S1C). We appreciate the reviewer’s insight that track length were surprisingly short, and after employing our drift correction and revised tracking methods, we now see substantially longer track lengths (Fig 2E, Fig 3C & F, Fig S2B & C). We additionally see a large fraction of tracks that persist throughout the imaging session (Fig 2E, Fig S2B & C).

(4) In Figure 3, the authors now quantify track length, but in this case in the unit 'minutes', from which I would interpret that this is now meant to assess the temporal dynamics rather than the spatial dynamics. The lack of a clear distinction between spatial dynamics and temporal dynamics is very confusing to me, since these are entirely independent measures. 'Track length' to me indicates spatial dynamics, and I would expect the units to be a measure of distance. 'Track duration', which the authors also use in some places, but inconsistently as far as I can tell, makes sense to me for the assessment of temporal dynamics, with the units being a measure of time. I would strongly recommend being very clear about this distinction, since the current representation of the data is very difficult to follow and interpret.

In addition to new spatial metrics, we have clarified in the text when we are referring to spatial dynamics (distance) versus temporal dynamics (time). As suggested, we use duration when referring to time, and speed or distance when referring to spatial metrics.

(5) The images from the newly generated CRISPR-based tags in Figures 5-7 are striking and very compelling - these will be very useful tools. However, here too, it seems that the interpretation of the data does not really match the results. All quantification indicates that there is very little change in synapse density or other assessed parameters over the time course of the imaging, and yet the authors emphasize the dynamic nature of visualized synapses. More compelling quantification would be needed to support this claim.

We have quantified spatial and temporal metrics for live neuron culture imaging for all tools developed including CRISPR-based tags (Figure 7).

(6) The discussion is extremely short and provides almost no integration of the results of the study into the framework of existing knowledge. Instead, it focuses almost exclusively on unanswered questions and future perspectives, which are also important, but not helpful in interpreting the findings from the current study. The latter aspects should be added to provide essential context for the current findings.

We agree and have added additional discussion of our current findings to help contextualize their significance.

We thank the Reviewers again for their positive feedback and insightful input, which has undoubtedly strengthened our study.
